# Yeast help identify cytopathic factors of Zika virus

**DOI:** 10.1186/s13578-017-0139-5

**Published:** 2017-02-20

**Authors:** Michael Bukrinsky

**Affiliations:** The George Washington University School of Medicine and Health Sciences, 2300 Eye St NW, Washington, DC 20037 USA

**Keywords:** Zika virus, ZIKV proteins, TOR pathway, Microcephaly, Yeast

## Abstract

Accumulating evidence implicates Zika virus (ZIKV) in pathogenesis of microcephaly in newborns and Guillain-Barré syndrome in adults. However, it remains unclear which viral proteins are responsible for these effects and what are the underlying mechanisms of their pathogenic activity. A recent paper by Drs. Zhao and Gallo, and their colleagues at University of Maryland in Baltimore used fission yeast for genome-wide analysis of ZIKV proteins. They demonstrated cytopathogenic activity for seven ZIKV proteins, anaC, C, prM, M, E, NS2B and NS4A. This activity was shown to be dependent on oxidative stress, and for NS4A they demonstrated involvement of the TOR stress-response pathway. Taken together, the findings presented in this paper provide the basis for further mechanistic studies that potentially can identify therapeutic means to treat neuro and immune complications of ZIKV infection.

The outbreak of Zika virus (ZIKV), a flavivirus initially identified in mid-twentieth century in Uganda, has now reached USA and has spread to other countries in North and South America where *Aedes* mosquitoes, which transmit the virus, inhabit [1]. The scary feature of ZIKV infection concerns its association with microcephaly and other birth defects in children born to infected mothers [2] and the Guillain-Barré syndrome caused by the immune system damaging the peripheral nerves in ZIKV-infected adults [3, 4]. This association is likely caused by ZIKV-induced damage of developing neurons (in microcephaly) and modification of peripheral nerves resulting in autoimmune reaction (in Guillain-Barré syndrome). Understanding the mechanisms of this damage is essential for developing therapeutic treatments, and the recent paper by Li et al. [5] makes an important step in this direction.

Study with primary human brain samples demonstrated that ZIKV infects neural stem cells, astrocytes, oligodendrocyte precursor cells, and microglia, whereas mature neurons were much less susceptible to infection [6]. These findings suggest that microcephaly in infants with congenital ZIKV infection may be caused by impairment of neural stem cell differentiation. Consistent with this mechanism, ZIKV was shown to increase cell death and dysregulate cell-cycle progression of neural progenitors [7] via activation of tall-like receptor (TLR) 3 [8]. However, the viral protein(s) responsible for this pathogenic activity remained unknown. Li et al. expressed all 14 structural and non-structural ZIKV proteins and peptides in fission yeast under an inducible promoter and analyzed the intracellular localization and cytopathic activities of each protein [5]. All but 5 proteins (Pr, NS1, NS3, 2K, and NS5) associated with intracellular membranes, mostly with endoplasmic reticulum (ER). Of these 9 membrane-associated proteins, 7 (anaC, C, prM, M, E, NS2B and NS4A) were found to exert cytopathic effects exhibited as defects in cellular proliferation, cell elongation and hypertrophy, nuclear fragmentation and cell cycle dysregulation, which resulted in cell death. Using a fluorescent dye specific for reactive oxygen species (ROS), the authors demonstrated strong fluorescence in cells expressing these seven proteins, suggesting that oxidative stress might be responsible for the observed cytopathic effect. Given that, at least in yeast, ROS induce Target of Rapamycin (TOR) cellular stress response [9], Li et al. followed up their observations by analyzing the effect of one of the ZIKV pathogenic proteins, NS4A, on yeast deleted in Tor1 (a key protein in the TOR pathway) or in Tip41 (negative regulator of the TOR pathway). Deletion of Tor1 abolished NS4A-induced cell hypertrophy and restored cell growth to normal. Expression of NS4A in Tip41-deleted cells worsened the NS4A-induced growth delay and cell hypertrophy, producing cells of spherical morphology. Surprisingly, the phenotype of these cells was similar to cells overexpressing Tip41, suggesting that NS4A may mimic the effect of NS4A, which inhibits the TOR signaling.

Results reported in the paper by Li et al. are provocative and raise new important questions. Is pathogenic activity of other ZIKV proteins also mediated by TOR? Is it mediated by TOR activation or inhibition? Is this mechanism relevant to human cells, and in particular to neural stem cells which are susceptible to ZIKV infection? What are the molecular mechanisms linking NS4A, and possibly other ZIKV proteins, to the TOR pathway? What is the connection between induction of ROS by ZIKV proteins and the effects on TOR? Some of these questions have been addressed in another report published almost at the same time as the paper by Li et al. [10]. In that report, the authors screened ZIKV proteins in human fetal neural stem cells and found that two proteins, NS4A and NS4B, inhibited mammalian TOR (mTOR) signaling by inhibiting phosphorylation of the Akt kinase, thus upregulating autophagy and impairing neurogenesis. Inhibition of Akt-mTOR signaling by ZIKV is consistent with the role of this pathway in cortical development [11], and provides a mechanistic link between ZIKV infection and microcephaly. These results suggest that other ZIKV proteins identified by Li et al. [5] exert their pathogenic activity via a different mechanism, e.g. induction of ROS.

## Abbreviations

ER: endoplasmic reticulum
ZKV: Zika virus
TLR: tall-like receptor
ROS: reactive oxygen species
TOR: Target of Rapamycin
mTOR: mammalian TOR
